# A Spontaneous Pneumomediastinum During an Asthma Exacerbation

**DOI:** 10.1002/rcr2.70156

**Published:** 2025-03-20

**Authors:** Joseph Derry, Harshana Bandara

**Affiliations:** ^1^ Northwest Lung Centre Wythenshawe Hospital, Manchester University NHS Foundation Trust Manchester UK

**Keywords:** air leak, asthma, pneumomediastinum, surgical emphysema

## Abstract

This case highlights the rare possibility of having pneumomediastinum in patients with asthma without having other structural lung disease. This also highlights the importance of vigilant reading of the chest x‐ray, including the proper inspection of the soft tissues areas.

## Graphical abstract

We report a rare case of Spontaneous Pneumomediastinum (SPM) in a patient with asthma presenting with severe retrosternal chest pain. This highlights the need for thorough imaging and a multidisciplinary approach. This also highlights the importance of vigilant reading of the chest x‐ray, including the proper inspection of the soft tissues areas.

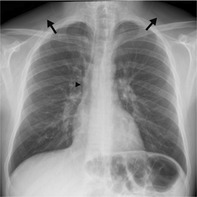

## 1 Clinical Image

A 23‐year‐old male with a childhood diagnosis of asthma, previously managed with inhalers but not on current treatment, presented with progressive shortness of breath and chest tightness starting mid‐flight from his holidays. He had been treated for a chest infection 3 weeks prior with oral antibiotics and steroids, which temporarily improved symptoms, but worsening breathlessness was noted during a one‐week holiday. He also reported new‐onset sore throat. He denied events of vomiting, retching, or long‐standing swallowing problems.

Social history revealed infrequent cigarette smoking, employment in a student union bar, and no exposure to pets or e‐cigarettes. He was allergic to amoxicillin. Examination revealed bilateral wheeze. He was treated with salbutamol nebulizers and hydrocortisone. On review by the respiratory team, a productive cough with yellow‐green sputum was noted, and treatment for a lower respiratory tract infection with oral doxycycline and prednisolone was initiated.

Laboratory findings included WCC 11.9 × 10^9^/L, neutrophils 9.93 × 10^9^/L, CRP 7 mg/L, and normal ECG findings. Observations remained stable, with PEF at 340 L/min. The diagnosis was revised to an acute asthma exacerbation, and nebulizers and systemic steroids were escalated.

**FIGURE 1:**
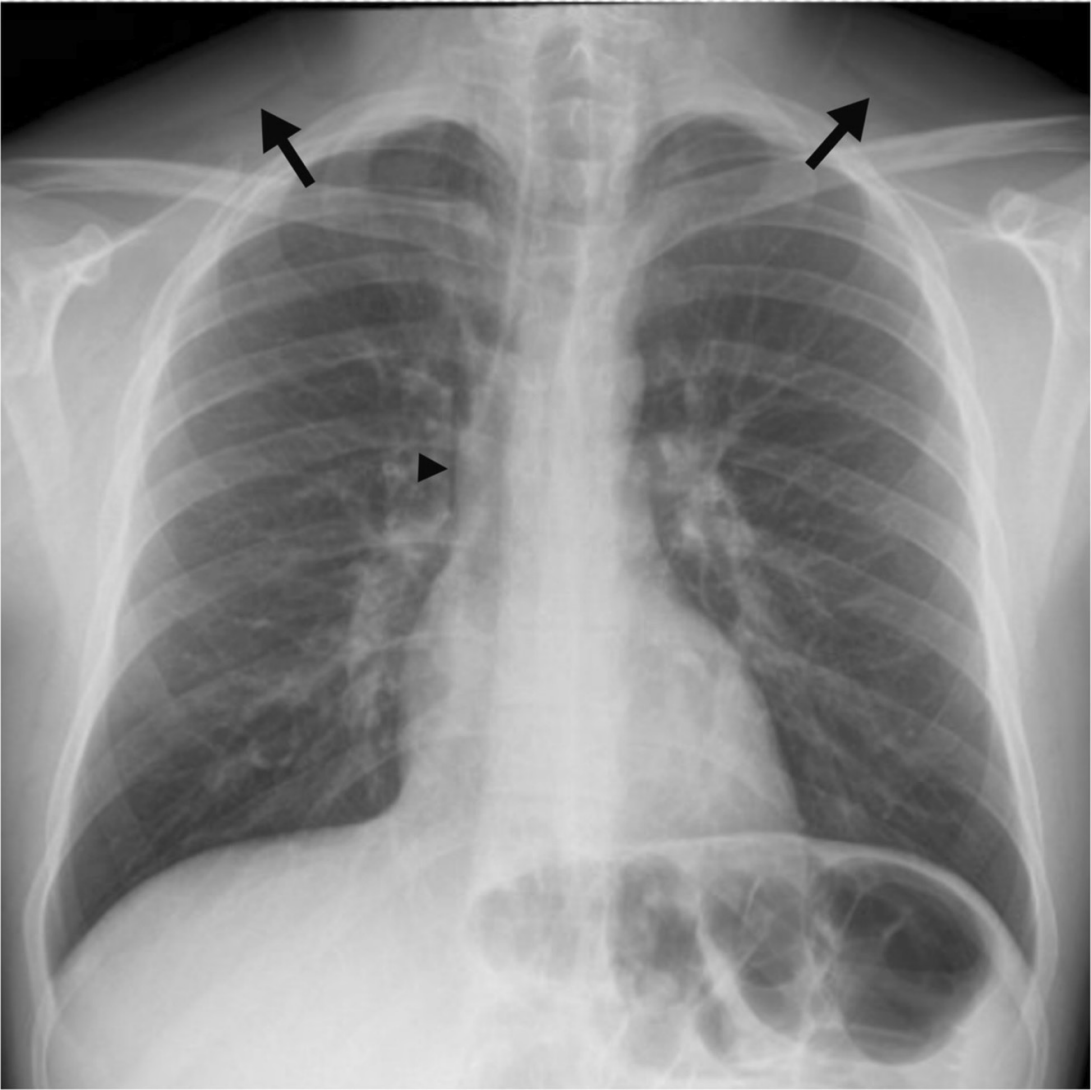
Admission posteroanterior CXR demonstrating subtle subcutaneous emphysema at the neck base (black arrow) and mediastinal free air at the right cardiac border (black arrowhead).

**FIGURE 2:**
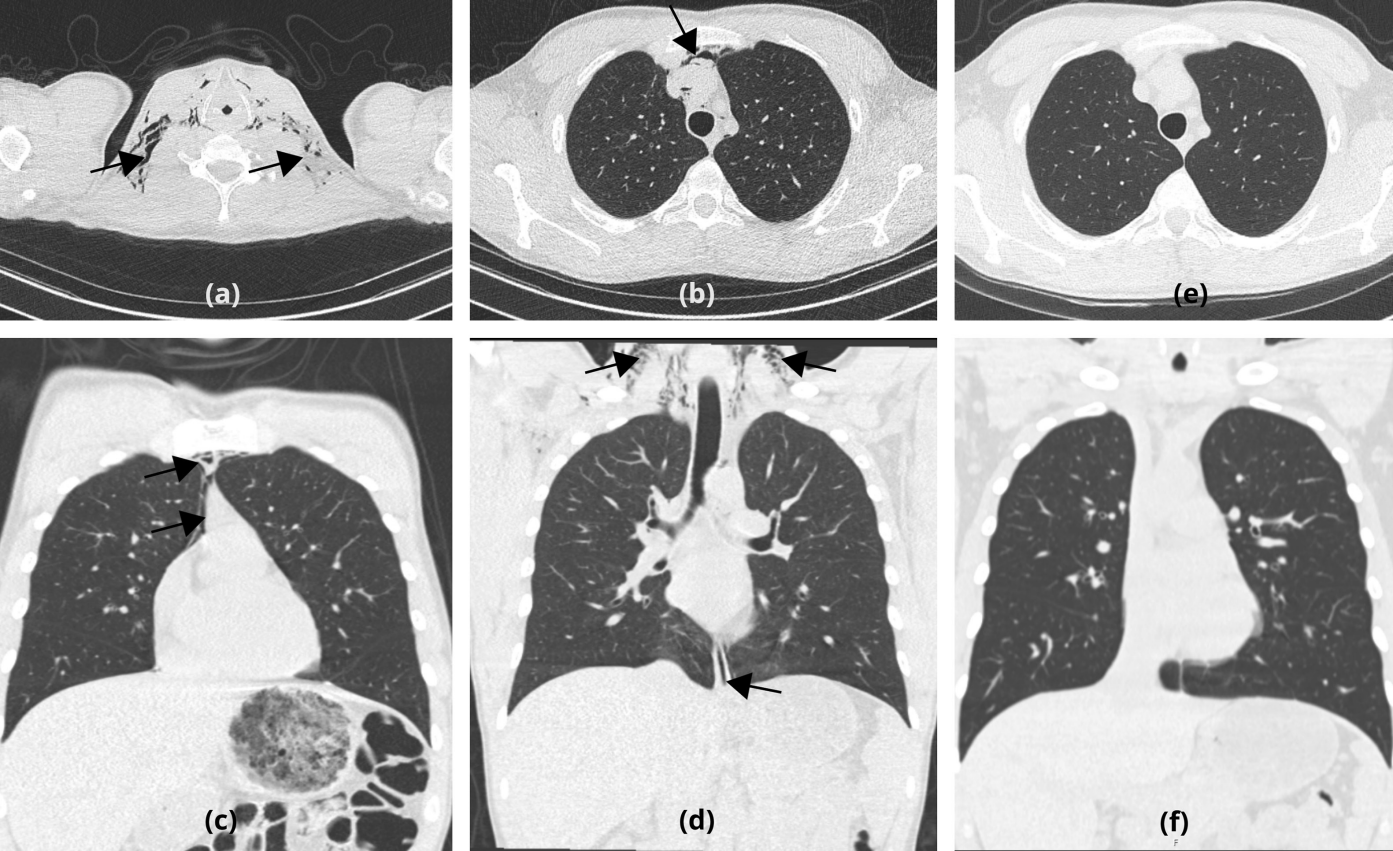
The initial CT images—The axial (a) and the coronal (d) images at the base of the neck showing the subcutaneous emphysema (black arrow), The axial images (b) and the coronal images (c, d) of the thorax showing the thin pneumomediastinum (black arrow). The follow‐up CT images in 4 weeks, demonstrating complete resolution of subcutaneous emphysema and pneumomediastinum (e and f) in both axial and coronal images.

The chest x‐ray (Figure 1) revealed evidence of subcutaneous emphysema around the base of the neck. CT thorax (Figure 2a–d) confirmed pneumomediastinum and stable subcutaneous emphysema, without evidence of pneumothorax. The oesophagus was also normal on the CT scan, and there were no lung parenchymal changes to suggest emphysema, cystic disease, or interstitial lung disease.

By Day 3, retrosternal pain and breathlessness had improved. A repeat CXR showed resolving subcutaneous emphysema, and nebulizers were reduced to as required. The case was discussed in the multi‐disciplinary team meeting, including a thoracic radiologist, and it was concluded that the SPM is likely secondary to asthma exacerbation and unlikely to be related to oesophageal rupture. The patient was discharged on Day 5 with a Maintenance and Reliever Therapy (MART) regime using a combined inhaler. Follow‐up included respiratory review with a follow‐up CT to confirm resolution of the pneumomediastinum (Figure 2e,f) and a gastroenterology review for any future upper gastrointestinal symptoms.

This case emphasises the importance of considering SPM, though rare, in patients with asthma presenting with severe retrosternal chest pain or unexplained symptoms, highlighting the need for thorough imaging and a multidisciplinary approach [1, 2]. This also highlights the importance of vigilant reading of the chest x‐ray, including the proper inspection of the soft tissues areas.

## Author Contributions

Joseph Derry drafted the manuscript. Harshana Bandara revised the manuscript. All authors approved the final manuscript.

## Ethics Statement

The authors declare that appropriate written informed consent was obtained for the publication of this manuscript and accompanying images.

## Conflicts of Interest

The authors declare no conflicts of interest.

## Data Availability

Data sharing is not applicable to this article as no new data were created or analysed in this study.
